# Antimetastatic effects of cordycepin mediated by the inhibition of mitochondrial activity and estrogen-related receptor α in human ovarian carcinoma cells

**DOI:** 10.18632/oncotarget.13829

**Published:** 2016-12-09

**Authors:** Chia-Woei Wang, Wei-Hsuan Hsu, Chen-Jei Tai

**Affiliations:** ^1^ Graduate Institute of Clinical Medicine, College of Medicine, Taipei Medical University, Taipei 11042, Taiwan; ^2^ Department of Obstetrics and Gynecology, School of Medicine, College of Medicine, Taipei Medical University, Taipei 11042, Taiwan; ^3^ Biochemical Process Technology Department, Center of Excellence for Drug Development, Biomedical Technology and Device Research Laboratories, Industrial Technology Research Institute, Hsinchu 30068, Taiwan; ^4^ Department of Traditional Chinese Medicine, Department of Internal Medicine, Taipei Medical University Hospital, Taipei 11042, Taiwan; ^5^ Traditional Herbal Medicine Research Center, Taipei Medical University Hospital, Taipei 11042, Taiwan

## Abstract

Cordycepin (3′-deoxyadenosine) is a compound for antitumor, which has been found to exert antiangiogenic, antimetastatic, and antiproliferative effects, as well as inducing apoptosis. However, the association between cancer metastasis and mitochondrial activity in cordycepin-treated ovarian carcinoma cells remains unclear. The 50 and 100 μM of cordycepin inhibits mitochondrial fusion and induces mitochondrial fission, respectively. These suggested that cordycepin showed the down-regulation of mitochondrial function and limitation of energy production. Because of activation of mitochondria and generation of energy are needed in cancer cell migration/invasion. After 24 h treatment, cordycepin suppresses epithelial–mesenchymal transition and migration in ovarian carcinoma cells through inhibiting estrogen-related receptor (ERR)-α. The ERRα is a co-transcription factor for gene expressions associated with mitochondrial fusion. Our results indicate that cordycepin suppresses metastasis and migration of ovarian carcinoma cells via inhibiting mitochondrial activity in non-toxic concentrations, and cordycepin has potential benefits in ovarian cancer therapy.

## INTRODUCTION

Ovarian cancer is a common gynecological cancer and is always found in woman worldwide. A high mortality rate is found in ovarian cancer patients when tumor invasion and metastasis. Clinically, onsurgical therapies such as chemotherapy or radiotherapy is always used to treat patients with ovarian cancer [1]. Ovarian cancer could be categorized into three subtypes, including (I) epithelial carcinomas, (II) stromal carcinomas, and (III) germ cell tumors [2], and the epithelial ovarian carcinomas is most found in patients in ovarian cancer cases [3, 4]. In addition, this ovarian epithelial tumor cells would result in migration/invasion through epithelial–mesenchymal transition (EMT) thereby entering into blood steam [5–8]. Several epithelial markers such as (I) epithelial keratins included E-cadherin, occludins, claudins, and desmoplakin are down-regulated and (II) acquire mesenchymal traits included vimentin, N-cadherin, fibronectin, and α-smooth muscle actin are up-regluated while development of EMT in cancer cells, these results will increase metastatic ability [9].

Cordycepin (3′-deoxyadenosine) is an antitumor compound isolated from Cordyceps. Recently, many studies have been reported that cordycepin shows antiangiogenic, antimetastatic, antiproliferative effects and apoptosis induction [10–14]. The association between migration and invasion and mitochondrial activity in cordycepin-treated ovarian carcinoma cells remains unclear, hence, cordycepin was tested for suppressing the migration and invasion of ovarian carcinoma cells and determined the inhibitory effects of cordycepin on the mitochondrial activity and EMT. Moreover, we have demonstrated that EMT and mitochondrial fusion induction were involved in metastasis in this study.

## RESULTS

### Cell viability and mitochondrial activity in cordycepin-treated OVCAR-3 cells

**Figure 1:**
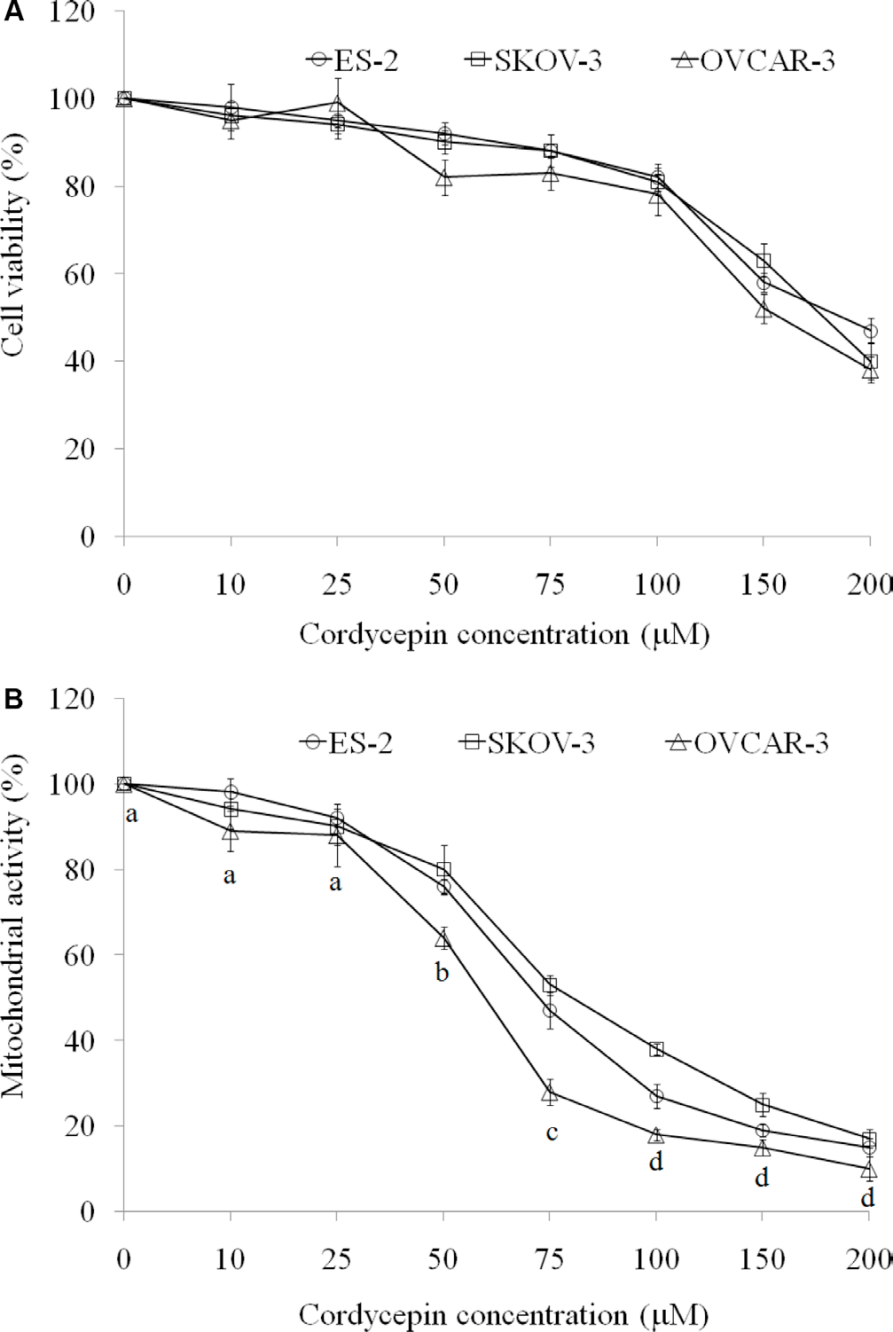
The effects of various concentration of cordycepin on (A) cell viability (crystal violet stain) and (B) mitochondrial activity (MTT assay) in the ES-2, SKOV-3, and OVCAR-3 human ovarian carcinoma cells after treatment for 24 h Data were shown as mean ± SD (*n* = 3). The statistical significance was evaluated and showed in OVCAR-3 cells treated with cordycepin.

Ovarian carcinoma cells (ES-2, SKOV-3, and OVCAR-3) were treated with cordycepin for 24 h; subsequently, cell viability was assessed through crystal violet staining method, which was not affected by mitochondrial interference [16]. Cell viability of ES- 2, SKOV-3, and OVCAR-3 cells were significantly decreased after treating with 150 or 200 μM cordycepin for 24 h while 10–100 μM cordycepin did not cause the cell death (Figure 1A).

3-(4,5-Dimethylthiazol-2-yl)-2,5-diphenyltetrazolium bromide (MTT) reduction is one of the most frequently used methods for measuring cell proliferation through the evaluation of mitochondrial activity. MTT reaction was used to investigate mitochondrial activity in ES-2, SKOV-3, and OVCAR-3 cells. Notably, 50–200 μM cordycepin markedly reduced the MTT reaction. In contrast to crystal violet staining, we considered cell death as the major reason for low MTT reaction at 150 or 200 μM of cordycepin treatment for 24 h. Hence, 50, 75, and 100 μM cordycepin should be noncytotoxic for attenuating mitochondrial activity (Figure 1B).

**Figure 2:**
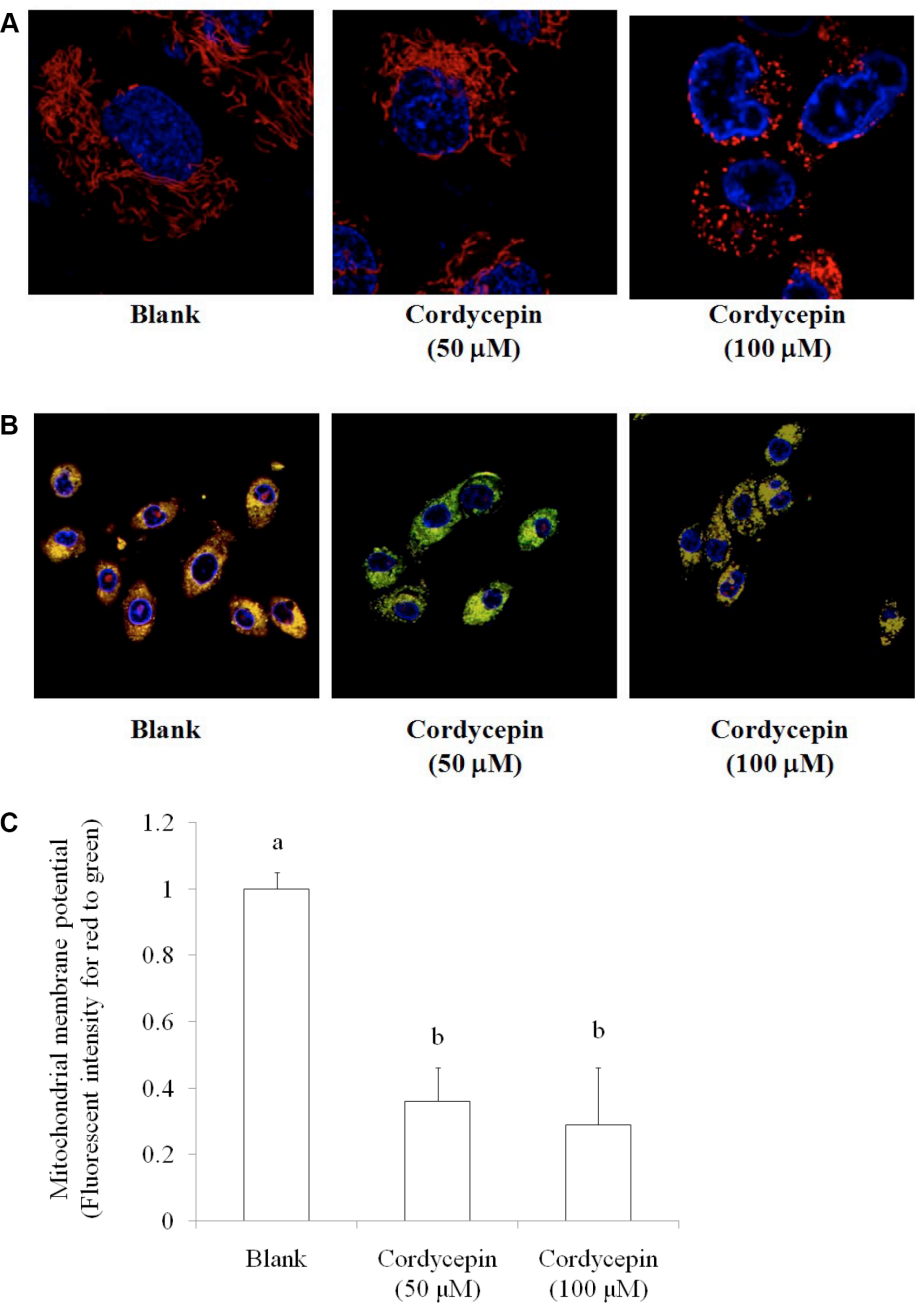
The effect of cordycepin (non-toxic dosage) on (**A**) mitochondrial morphology stained by MitoTracker Deep Red-FM and (**B**) mitochondrial membrane potential stained by JC-1 in the OVCAR-3 ovarian carcinoma cells after 24 h treatment. (**C**) The statistical significance was evaluated and showed in mitochondrial membrane potential. Data were shown as mean ± SD (*n* = 3).

In the MTT assay, both mitochondrial morphology and membrane potential are indices for mitochondrial function. Fission and fusion are need to have balance for regulations of cell growth, mitochondrial redistribution, and energy production. These circumstances plays important roles in apoptosis and mitophagy [16]. Data showed that treating with 50 and 100 μM cordycepin changed the mitochondrial distribution and induced mitochondrial fission, respectively (Figure 2A). Mitochondrial membrane potential is a crucial parameter of mitochondrial function that is used as an indicator of cell health. JC-1 is a lipophilic, cationic dye that can selectively enter mitochondria and reversibly change its color from green to red with increasing membrane potential. In healthy cells with high levels of mitochondria, JC-1 spontaneously forms complexes known as J-aggregates, with intense red fluorescence. By contrast, in apoptotic or unhealthy cells with low mitochondrial membrane potential, JC-1 remains in the monomeric form, which shows only green fluorescence. Figure 2B indicated that 50 and 100 μM cordycepin treatment markedly decreased the mitochondrial membrane potential.

### Effects of cordycepin on EMT and mitochondria fusion of OVCAR-3 cells

**Figure 3:**
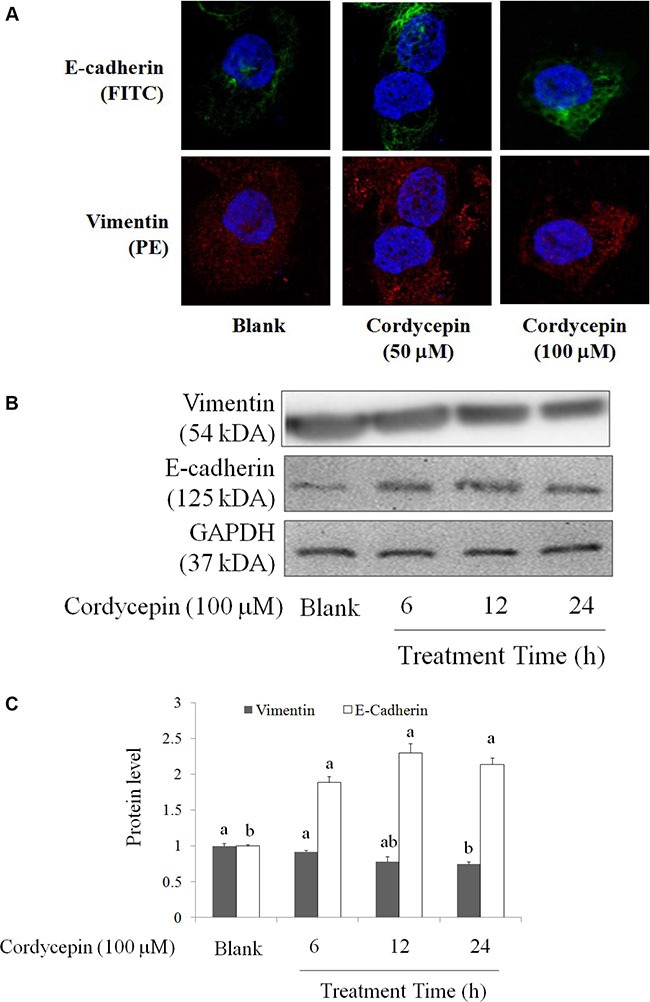
The regulations of cordycepin on EMT markers of the OVCAR-3 ovarian carcinoma cells after 24 h treatment, including E-cadherin (FITC) and vimentin (PE) by (**A**) fluorescent microscopy and (**B**) Western blot. Data were shown as mean ± SD (*n* = 3). ^a.b^ values with one different letter superscript are significantly different from each other (*p* < 0.05).

EMT is a major mechanism involved in cancer metastasis [17], it also triggers cell proliferation and drug resistance [18, 19]. Therefore, inhibition of EMT mediated by a novel medicine is a complementary therapeutic method for suppressions of metastasis and chemotherapy resistance [19]. 50 and 100 μM cordycepin treatment for 24 h markedly increased E-cadherin level and decreased vimentin level in OVCAR-3 cells (Figure 3).

**Figure 4:**
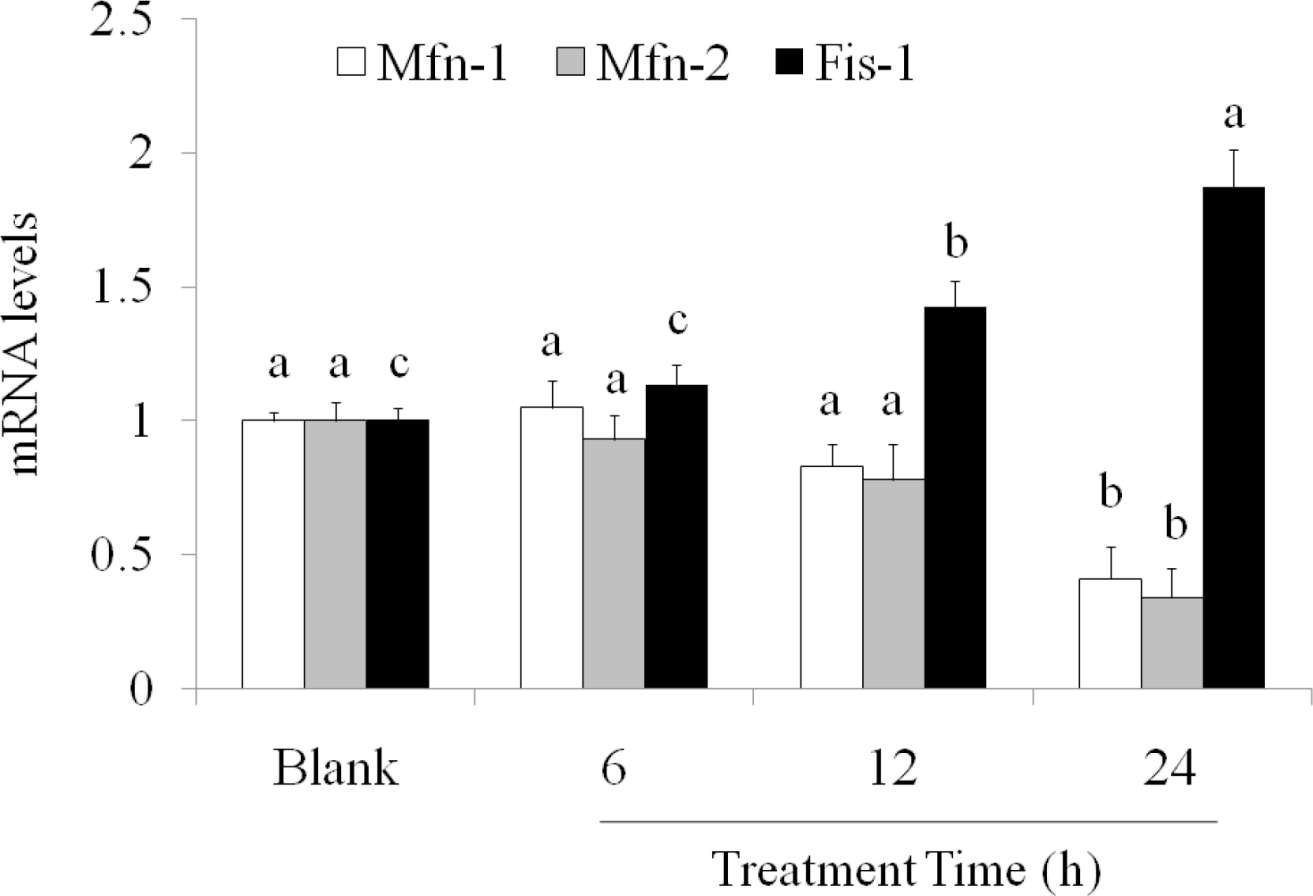
Effects of cordycepin on (**A**) Mfn1, (**B**) Mfn2, and (**C**) Fis1 mRNA levels in the OVCAR-3 ovarian carcinoma cells after 24 h treatment. Data were shown as mean ± SD (*n* = 3). ^a.b^ values with one different letter superscript are significantly different from each other (*p* < 0.05).

Mitochondrial fusion or fission state is accorded to mitochondrial division. Mitofusin (Mfn)-1 and -2 (Mfn-1/2), and GTPase optic atrophy-1 are found to link two mitochondria for fusion. By contrast, dynamin-related protein-1 and fission protein (Fis)-1 separate mitochondria for fission [20, 21]. Cordycepin (100 μM) significantly suppressed the mRNA levels of Mfn-1 and Mfn-2 in OVCAR-3 cells. By contrast, after 24 h treatment, 100 μM of cordycepin elevated the mRNA level of Fis-1 (Figure 4).

### Effects of cordycepin on the migration in OVCAR-3 cells

**Figure 5:**
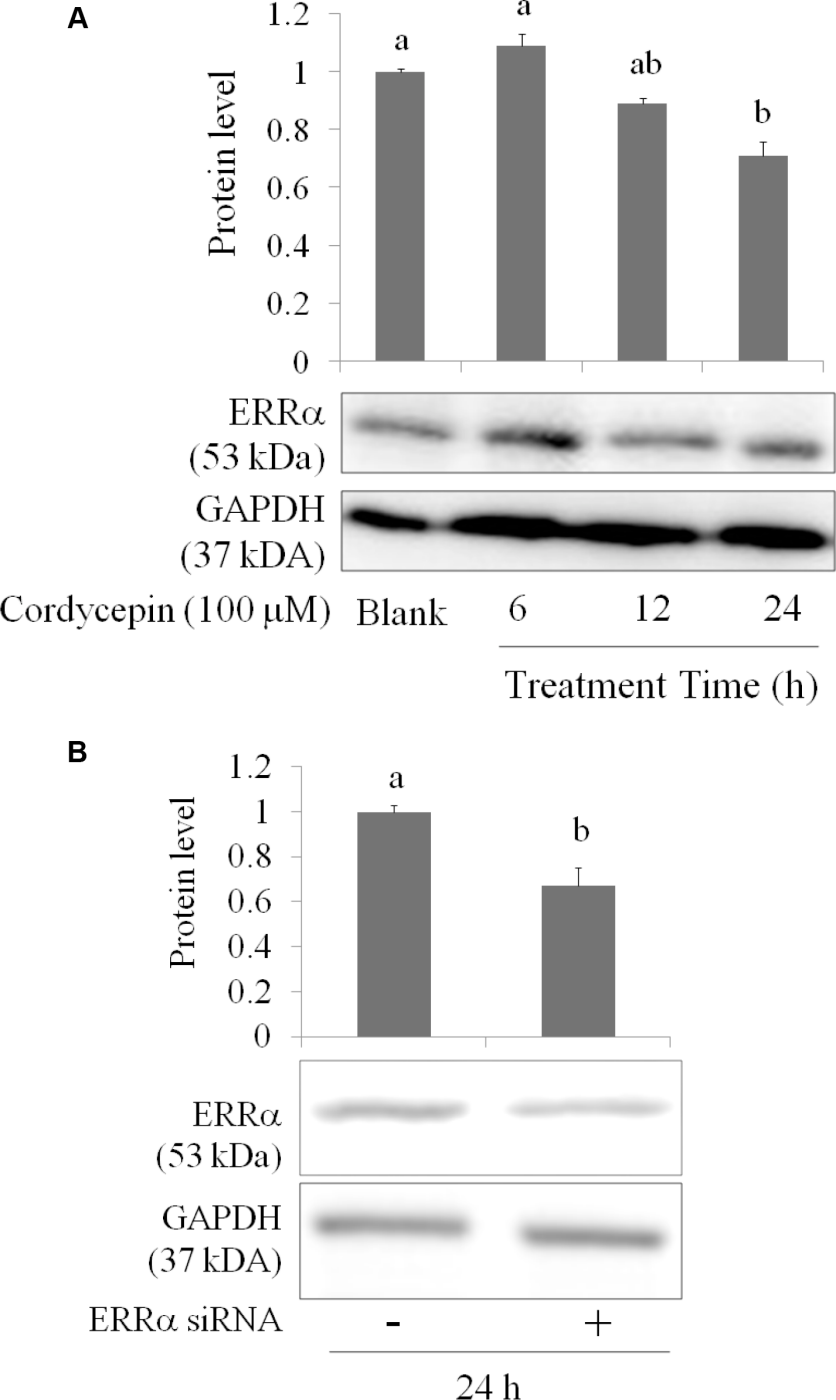
(**A**) The inhibitory effects of cordycepin on ERRα level of the OVCAR-3 ovarian carcinoma cells after 24 h treatment. (**B**) The attenuation of ERRα siRNA for 12 h treatment in the OVCAR-3 ovarian carcinoma cells. Data were shown as mean ± SD (n = 3). ^a.b^ values with one different letter superscript are significantly different from each other (*p* < 0.05).

Transcription factor ERRα regulates mitochondrial function and cancer cell viability [22]. ERRα promotes mitochondrial fusion via elevating Mfn-1 and Mfn-2 proteins [21]. In addition, ERRα suppression results in EMT inhibition in breast cancer cells [23]. Metastasis, proliferation, and migration of A549 lung cancer cells were increased when ERRα overexpression [24]. We speculate that cordycepin inhibits mitochondrial activity and EMT in OVCAR-3 cells may due to ERRα regulation. Indeed, ERRα expression was decreased after cordycepin treatment at 100 μM for 24 h (Figure 5A). We used specific small interfering (si) RNA for ERRα in OVCAR-3 cells to confirm our results (Figure 5B).

**Figure 6:**
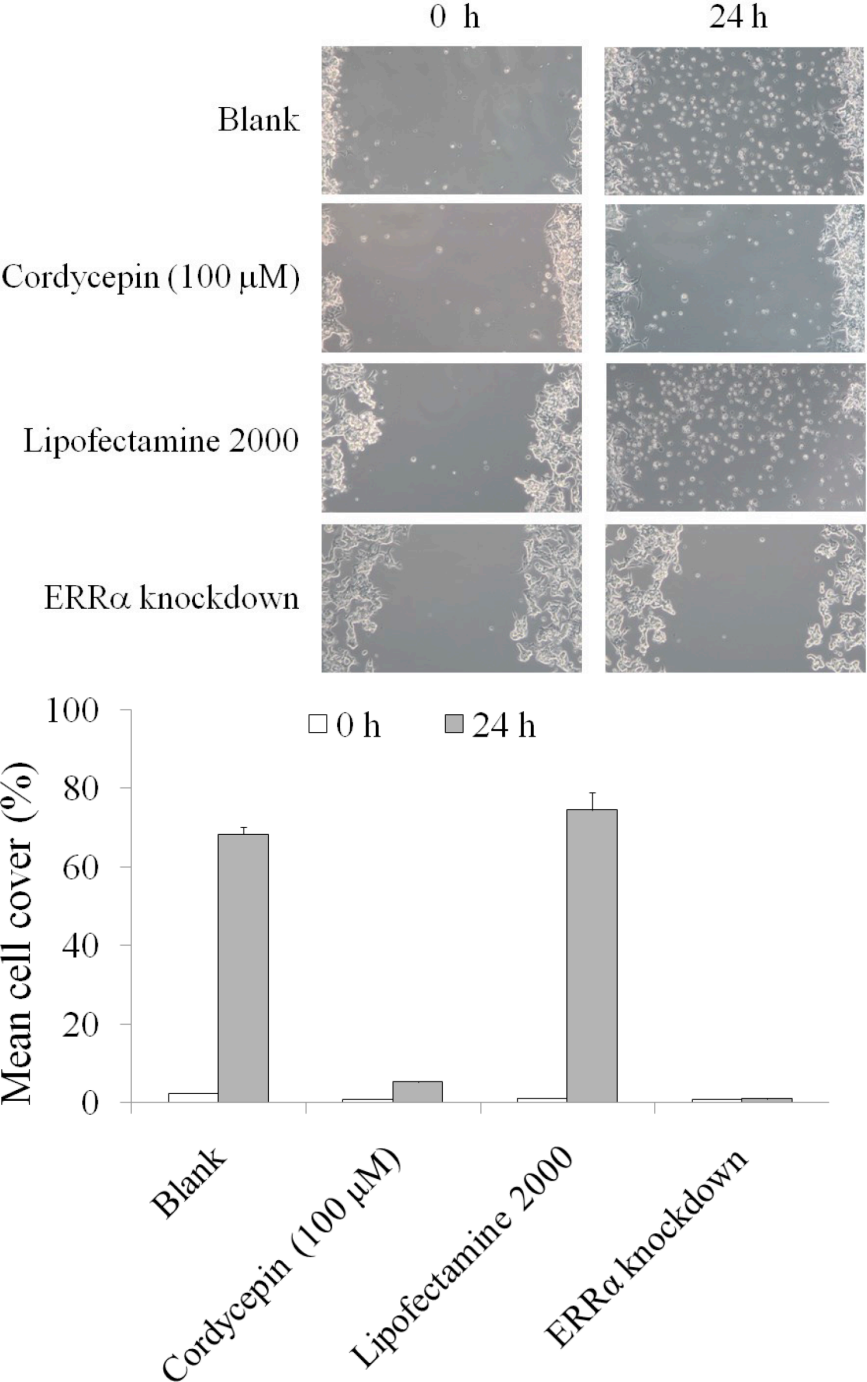
The inhibitory effects of migration in the OVCAR-3 human ovarian carcinoma cells treated by cordycepin for 24 h

We subsequently investigated the migration of OVCAR-3 cells after ERRα knockdown and cordycepin treatment. As illustrated in Figure 6, cordycepin treatment for 24 h significantly suppressed the migration of OVCAR-3 cells; this result was similar to that obtained after ERRα knockdown. Collectively, the data revealed that cordycepin inhibited EMT and migration in OVCAR-3 cell by inducing mitochondrial fission and suppressing the mitochondrial membrane potential, and then decreasing mitochondrial activity.

## DISCUSSION

Because of chemotherapy resistance and metastasis, many complementary and alternative medicine are developed in applications of cancer prevention and therapy. Traditional Chinese medicine is one of treatment for complementary and alternative therapy. Cordycepin is an active compound and has been used in cancer treatment in past clinical study.

Loss of intercellular junctions and raise of cell motility are casue by EMT in cancer cell, thereby resuting in metastasis [25]. The E-cadherin is an adhesion molecules on cell membrane, which expression is going to affect cell junction. A study has reported that reduction of E-cadherin is an target index for first stage of cancer cell metastasis [26, 27]. Subsequently, other biomarkers are also change involved in EMT, including N-cadherin, fibronectin, and vimentin [27, 28], leading to cancer cell migration and invasion [28–30]. We recently reported that EMT upregulation was induced by mitochondrial fusion in ovarian carcinoma cells [21].

Mitochondria undergo fission and fusion change called mitochondrial dynamics, which regulates cell metabolism, survival, and proliferation [31]. Fusion unifies the mitochondrial compartments, whereas fission generates morphologically and functionally distinct mitochondria. Mitochondrial fission often occurs early in an apoptotic event [32] and the autophagic process [33]. Mitochondrial fusion is associated with increased cell survival [34].

**Figure 7:**
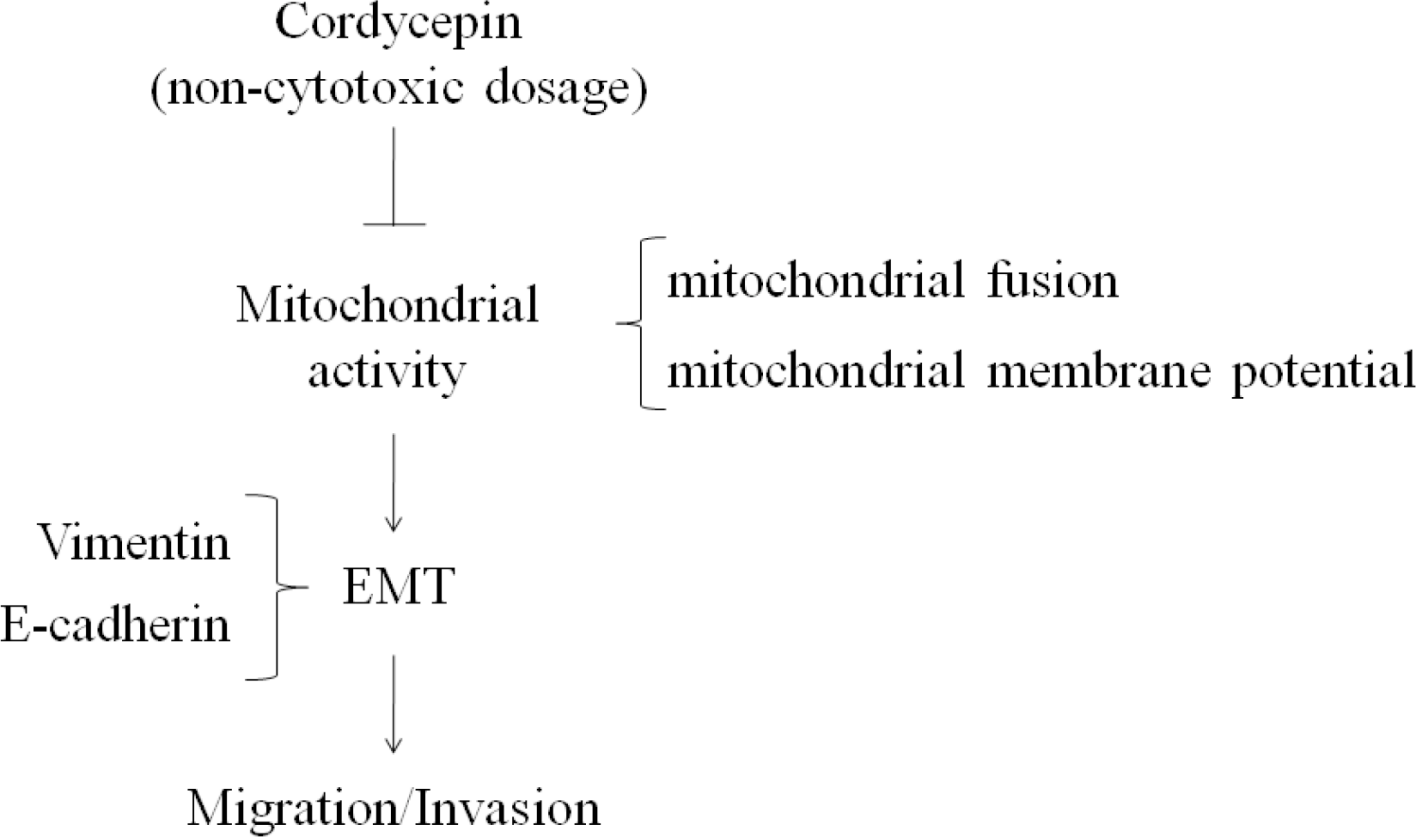
The anti-invasion of cordycepin in ovarian carcinoma mediated by down-regulation of mitochondrial activity

Our results suggest that ERRα elevated Mfn-1 and Mfn-2 to induce EMT, resulting in cancer cell migration. However, ERRα expression and EMT (E-cadherin loss and vimentin elevation) in OVCAR-3 cells were inhibited by noncytotoxic concentration of cordycepin (100 μM) treatment. Cordycepin suppressed mitochondrial activity, thereby avoiding EMT and ovarian carcinoma cells migration (Figure 7). This is the first study to confirm the inhibition of metastasis and migration in cordycepin-treated ovarian carcinoma cells. Moreover, our results suggested that cordycepin inhibited mitochondrial activity at noncytotoxic concentrations and suppressed ovarian carcinoma cells migration.

## MATERIALS AND METHODS

### Chemicals

Crystal violet, sodium dodecyl sulfate (SDS), Triton X-100, trypsin, cordycepin, and trypan blue were purchased from Sigma Chemical Co. (St. Louis, MO, USA). Fetal bovine serum (FBS) was purchased from Life Technologies (Auckland, New Zealand). Dimethyl sulfoxide was purchased from Wako Pure Chemical Industries (Saitama, Japan). MitoTracker Deep Red-FM was purchased from Invitrogen (Carlsbad, CA, USA). Anti-vimentin and -ERRα antibodies were purchased from Santa Cruz (Santa Cruz, CA, USA). Anti-E-cadherin antibody was purchased from Abcam (CA, USA).

### Cell culture

Human ovarian carcinoma cell lines (ES-2, SKOV-3, and OVCAR-3) were grown in Dulbecco’s modified Eagle medium (Gibco BRL, Grand Island, NY, USA) containing 2 mM L-glutamine and 1.5 g/L of sodium bicarbonate, supplemented with 10% FBS (Gibco BRL) and 2% penicillin–streptomycin (10,000 U/mL of penicillin and 10 mg/mL of streptomycin). The cells were cultured in a humidified incubator at 37°C under 5% CO_2_.

### Cell viability

The cytotoxic effect of cordycepin on ovarian carcinoma cells was measured using crystal violet staining assay. The cells were seeded on 24-well plates (3 × 10^4^ cells/well) and treated with various concentrations of cordycepin for 24 h. The cells were subsequently washed and stained with 2 g/L of crystal violet in phosphate-buffered formaldehyde for 20 min. Crystal violet bound to the cells was dissolved in 20 g/L of SDS solution, and the corresponding absorbance was measured at 600 nm [16].

### MTT assay

The mitochondrial activity of cordycepin in ovarian carcinoma cells was measured using the MTT staining assay. The cells were seeded on 24-well plates (3 × 10^4^ cells/well) and treated with various concentrations of cordycepin for 24 h. The cells were reacted with MTT (0.5 mg/mL) for 2 h and then washed, after which the tetrazolium was dissolved with dimethyl sulfoxide, the absorbance was measured at 570 nm.

### Western blot

Cells were rinsed and lysed using RIPA buffer with protease and phosphatase inhibitors for 20 min on ice. The cells were then centrifuged at 12,000 ×g for 10 min at 4°C. Protein extracts were resolved through SDS–polyacrylamide gel electrophoresis. The protein bands were electrotransferred to nitrocellulose membranes and treated with enhanced chemiluminescence (ECL) blocking agent (GE Healthcare Bio-Sciences) in saline buffer (T-TBS) for 1 h and then incubated with a primary antibody overnight at 4°C. Subsequently, the membranes were washed three times in T-TBS, and the bound antibodies were detected using appropriate horseradish peroxidase-conjugated secondary antibodies, followed by analysis in an ECL plus Western blotting detection system (GE Healthcare Bio-Sciences).

### Mitochondrial morphology and mitochondrial membrane potential

Cells were treated with 250 nM Mitotracker Deep-Red FM (Invitrogen) for 30 min in a serum-free culture medium to determine the mitochondrial morphology. After washed the cells, the nuclei were stained with Hochest 33342 [21]. The mitochondrial morphology was observed using a confocal microscope. Mitochondrial membrane potential was measured by JC-1 staining, and the flourscent intensity was determined through microscopy.

### ERRα knockdown

ERRα interference in OVCAR-3 cells was determined using a lipofectamine RNAiMAX transfection reagent, according to the manufacturer’s protocols (Invitrogen). The sequence of specific siRNAs for ERRα was purchased from Santa Cruz (Santa Cruz). Cell lysates were subjected to Western blotting with an ERRα antibody to confirm the inhibition of ERRα expression.

### Real-time PCR

Total RNA was obtained using the Trizol reagent (Gibco BRL Life Technologies, Inc., Gaithersburg, MD, USA), according to the manufacturer’s instructions. Primers were synthesized by MD-Bio Inc. (Taipei, Taiwan). The gene expression level was determined through relative quantitative real-time polymerase chain reaction (CFX Cycler System, Bio-Rad Laboratories, Inc., Hercules, CA, USA).

### Statistical analysis

The statistical significance was determined by one-way analysis of variance (ANOVA) using the general linear model procedure of SPSS software (SPSS Institute, Inc., Chicago, IL, USA), followed by ANOVA with Duncan’s test. The results were considered to be statistically significant if the *p value* was < 0.05.

## CONCLUSIONS

Cordycepin is an active compound in various *Cordyceps* spp. and widely used in traditional Chinese medicine. It can considerably inhibit human ovarian carcinoma cells by suppressing EMT and mitochondrial activity through ERRα inhibition. Our results indicate that cordycepin has potential benefits for anti-metastasis and anti-migration in ovarian cancer therapy.
